# In recreational alpine skiing, the ACL is predominantly injured in all knee injuries needing hospitalisation

**DOI:** 10.1007/s00167-020-06221-z

**Published:** 2020-08-14

**Authors:** Markus Posch, Alois Schranz, Manfred Lener, Katja Tecklenburg, Martin Burtscher, Gerhard Ruedl

**Affiliations:** 1 Department of Sport Science of the University of Innsbruck, 6020 Innsbruck, Austria; 2 medalp sportclinic, 6460 Imst, Austria

**Keywords:** Recreational alpine skiing, Knee injury pattern, ACL, Concomitant injuries, Epidemiology

## Abstract

**Purpose:**

The knee joint still represents the most frequent anatomical injury location accounting for about one-third of all injuries in recreational alpine skiers. However, comprehensive information on current knee injury patterns in this populations is sparse.

**Methods:**

During the winter seasons 2016/17 and 2019/20, this retrospective questionnaire-based study was conducted in an Austrian sportclinic situated in a large ski area. Among a cohort of 282 recreational skiers (51.8% females), all injuries were diagnosed by the use of magnetic resonance imaging. Additionally, data were recorded on anthropometric characteristics, the perceived speed at the moment of injury, type of fall, physical fitness, self-reported skill level and risk-taking behaviour.

**Results:**

The anterior cruciate ligament (ACL) was injured in all knee injuries recorded. Of the total study sample, 64.5% (*n* = 182) were ACL injuries with concomitant injuries and about 35.5% (*n* = 100) were isolated ACL injuries, not involving any other structures of the knee joint. In general, most common concomitant injury diagnoses among ACL-injured recreational alpine skiers were injuries of the medial collateral ligament (MCL) (*n* = 92, 50.5%), medial meniscus (MM) (*n* = 73, 40.1%) and lateral collateral ligament (LCL) (*n* = 41, 22.5%). No significant differences regarding additionally recorded characteristics were found between ACL-injured individuals with concomitant injuries and those with isolated ACL injury.

**Conclusions:**

Whereas, before the introduction of carving skis, the MCL was reported being the most common injured part of the knee, currently, the majority of knee injuries are ACL injuries accompanied by injury of other knee joint structures, i.e. the MCL, MM and LCL.

**Level of evidence:**

Level III.

## Introduction

Recreational alpine (downhill) skiing represents one of the most popular winter sports, enjoyed by several 100 million people annually [1, 2]. However, alpine skiing is also associated with a certain risk for injury [3, 4].

The overall incidence of ski injuries among recreational skiers has decreased from 5–8 injuries per 1000 skier days before the 1970s [5], to 2–3 injuries per 1000 skier days in the early 1990s [6, 7]. The actually evaluated injury rate in Austria is even less than one injury per 1000 skier days [8]. Despite the steady downward trend in alpine skiing injuries, it is noticeable that the proportion of knee injuries did not benefit from this decline to the same amount [9]. Burtscher et al. [10] showed that with the introduction of the short and shaped carving skis, the overall injury rate has even decreased by 9%.

The knee joint still represents the most frequent anatomical injury location accounting for about one-third of all injuries in recreational alpine skiers [8, 10, 11]. Currently, the most common knee injury in recreational alpine skiers is a rupture of the anterior cruciate ligament (ACL) with 15–21% of all injuries in adult skiers of both sexes [4, 12].

Prior to the introduction of the short and shaped carving skis in the late 90s, several studies reported a sprain of the medial collateral ligament (MCL) being the most common knee injury [7, 13]. As these MCL injuries in skiers were mostly grade I or grade II sprains and were, therefore, treated conservatively [14, 15], ACL injuries were paramount [16]. According to studies that included skiers using so-called traditional, long and unshaped skis, ACL-injured skiers sustained a high incidence of concomitant injuries of the knee joint [7, 14, 15, 17, 18]. In a study by Duncan et al. [18], 68% of ACL tears were combined with other injuries. Moreover, injuries of the menisci associated with ACL ruptures ranged from 23 to 55% [14, 18]. Studies by Rust et al. [19] and Wölfel et al. [20] demonstrated that among skiers using so-called carving skis (short and shaped), an ACL rupture was the most frequent knee injury diagnosis among all skiing-related injuries (16.7% and 15.8%). However, as no detailed information about the injury diagnosis was provided and no standardised rating of the severity of the injury was applied [19–21], it remains unknown whether knee injury patterns among recreational alpine skiers have changed in the last years.

In general, literature concerning current knee injury patterns and injury characteristics among knee-injured recreational skiers is sparse but could help to understand injury mechanisms and to improve preventive measures to potentially avoid complex knee injuries in recreational alpine skiing. The study by Granan et al. [22] focused on investigating injury patterns at the time of ACL reconstructions and does not provide an overview of current knee injury patterns of injured recreational skiers in general. Another recent study by Rust et al. [19] explored the influence on the addition of snowboarders to a large ski resort on a potential change in both the rate and pattern of injuries treated and does not give detailed information about diagnosis and severity of knee injuries. To the best of our knowledge, there is no study reporting detailed knee injury patterns and concomitant injuries among recreational alpine skiers. It was hypothesised that knee injury patterns and concomitant injuries in recreational alpine skiing have changed during the last decades.

Therefore, the purpose of this study was to analyse comprehensive current data on knee-injured recreational skiers during the winter seasons 2016/17 and 2019/20.

## Materials and methods

This study was conducted as a retrospective questionnaire-based study during the winter seasons 2016/17 and 2019/20 in an Austrian sportclinic situated in a large ski area. The survey was conducted according to the ethical guidelines for surveys approved by the Institutional Review Board (IRB) as well as the Board for Ethical Issues (BfEI) of the University of Innsbruck (25/2016).

Inclusion criteria were all (non-contact) knee injuries and related concomitant injuries after a self-inflicted fall, an age > 17 years, and the use of any type of carving skis (in contrast to long and unshaped traditional skis as well as to so-called short ski boards).

Similar to a study by Posch et al. [23], patients were interviewed between the months December and April (20 days on average per season) using a predefined standardised questionnaire.

The random recruitment of patients was dependent upon logistical aspects at the sportclinic (availability of rooms and personnel) and willingness of patients to volunteer [24]. More than 95% of invited patients agreed to participate in the study. All study participants were informed about the aims of the study and gave their written informed consent for participating.

### Injury diagnosis

In accordance with the previous studies by Posch et al. [23, 25], all types of knee injuries and related concomitant injuries were diagnosed by a physician present using magnetic resonance imaging (MRI), which is directly located in the sportclinic.

To analyse the frequency of concomitant knee injuries the following injury locations were used/defined/categorised: Anterior Cruciate Ligament (ACL), Medial Collateral Ligament (MCL), Lateral Collateral Ligament (LCL), Lateral Meniscus (LM), Medial Meniscus (MM), and Cartilage.

Furthermore, to enable a standardised definition of the injury severity, knee injuries to the ACL, MCL, LCL, LM, MM and Cartilage were rated by the physician as partial (grade II) or complete tear (grade III) [26].

Patients who suffered an ACL injury without any concomitant injuries to the MCL, LCL, LM, MM and cartilage were defined as isolated ACL injuries.

According to O`Donoghue`s [27], the classic unhappy triad was defined as injuries to the ACL, MCL and MM.

### Questionnaire

Similar to the questionnaires used in recent studies by Posch et al. [23, 25], Ruedl et al. [26], Burtscher et al. [10, 28], knee-injured (recreational alpine) skiers were asked on age, sex, height, weight, perceived speed at the moment of injury (very fast, fast, moderate, slow, very slow), type of fall (forward fall with and without body rotation, backward fall with an without body rotation), physical fitness (very good, good, moderate, poor). Moreover, study participants had to self-report their skill level (expert, advanced, intermediate and beginner) according to Sulheim et al. [29] and risk-taking behaviour (more risky vs. more cautious) according to Ruedl et al. [30]. In addition, participants were divided into more skilled (expert and advanced) and into less skilled (intermediate and beginner) skiers as a tendency was shown to underestimate individual skiing skills, especially among female skiers [29].

### Statistical analysis

All data are presented as means, absolute and relative frequencies. According to the tests on normal distribution (Kolmogorov–Smirnov), univariate differences among metric data [mean age, body mass index (BMI)] between the two groups (isolated ACL injury vs. ACL injury and concomitant injuries) were evaluated by independent *t*-tests or Mann–Whitney-*U* tests. Differences in frequencies (perceived speed, type of fall, physical fitness, skill level, risk taking behaviour) were evaluated by Chi-square tests. SPSS 23.0 (IBM Corporation, Armonk, NY) was used for the statistical analysis. All *p*-values were two-tailed and statistical differences were considered significant at *p* < 0.05.

## Results

Comprehensive data sets from 282 knee-injured recreational alpine skiers (48.2% males, 51.8% females) with a mean age of 44.0 ± 10.1 years, mean height of 1.73 ± 0.08 m and mean weight of 72.3 ± 10.3 kg have been analysed in the present study.

All analysed knee injuries in this study always involved an injury to the ACL. Of the total study sample, 64.5% (*n* = 182) were injuries to the ACL with concomitant injuries and about 35.5% (*n* = 100) were isolated ACL injuries, not involving any other structure of the knee joint.

In total, 35.1% of all ACL injuries, isolated and combined ACL injuries, were grade II, describing a partial rupture and 64.9% were grade III, indicating a complete tear.

In general, most common concomitant injury diagnoses among knee-injured recreational alpine skiers were an injury to the MCL (*n* = 92, 50.5%), MM (*n* = 73, 40.1%) and LCL (*n* = 41, 22.5%). All concomitant injuries were mainly grade II (MCL 75.0%, MM 94.5%, LCL 70.7%).

No injury to the PCL was observed and only four skiers suffered from cartilage damage. Three patients reported a previous injury of the contralateral knee joint.

O`Donoghues`s [27] classic unhappy triad of injuries to the ACL, MCL and MM was seen 7 times.

No significant differences were found among sex, age, BMI, physical fitness, skill level, risk taking behaviour, perceived speed and type of fall between skiers with an ACL injury and concomitant injuries and skiers with an isolated ACL injury (Table 1).

**Table 1:** Characteristics of knee injuries among recreational alpine skiers with a knee injury between winter seasons 2016/17 and 2019/20

	ACL injuries with concomitant injuries (*n* = 182)	Isolated ACL injury (*n* = 100)	*p* value
Sex *n* (%)			
Male	83 (45.6)	53 (53.0)	
Female	99 (54.4)	47 (47.0)	n.s.
Age (mean ± SD)	43.1 ± 9.6	45.5 ± 10.7	n.s.
Body Mass Index (mean ± SD)	24.0 ± 2.3	24.3 ± 2.3	n.s.
Physical fitness *n* (%)			n.s.
Very good	32 (17.6)	15 (15.0)	
Good	102 (56.0)	62 (62.0)	
Moderate	43 (23.7)	20 (20.0)	
Poor	5 (2.7)	3 (3.0)	
Skill level *n* (%)			n.s.
More skilled	93 (51.1)	55 (55.0)	
Less skilled	89 (48.9)	45 (45.0)	
Risk taking behaviour *n* (%)			n.s.
More cautious	106 (58.2)	47 (47.0)	
More risky	76 (41.8)	53 (53.0)	
Perceived speed at the time of injury *n* (%)			n.s.
Very fast	3 (1.6)	2 (2.0)	
Fast	56 (30.8)	30 (30.0)	
Moderate	56 (30.8)	35 (35.0)	
Slow	48 (26.4)	20 (20.0)	
Very slow	19 (10.4)	13 (13.0)	
Type of fall *n* (%)			n.s.
Forward fall with body rotation	126 (69.2)	59 (59.0)	
Forward fall without body rotation	17 (9.3)	15 (15.0)	
Backward fall with body rotation	39 (21.4)	26 (26.0)	
Backward fall without body rotation	/	/	

Data are presented as mean ± SD, absolute and relative frequencies

## Discussion

The most important finding of the present study was that all knee injuries involved an injury to the ACL and the majority of knee injuries were ACL injuries with concomitant injuries of other knee joint structures, i.e. injuries to the MCL, MM and LCL.

In contrast to earlier studies [7, 13] which reported the MCL being the most injured part of the knee, with regard to ACL injuries, this study shows that the ACL is predominantly affected and frequently accompanied by injuries of other knee joint structures. A possible reason for this discrepancy might be that all patients of the present study used typical short and shaped carving skis; whereas in earlier studies of Warme et al. [7] and Johnson and Ettlinger [13], subjects used traditional (long) skis. Carving skis are more tailed than traditional skis and therefore, it seems easier to catch an edge resulting in a forward twisting fall favouring an ACL injury [31]. Moreover, Köhne et al. [32] reported that catching the edge causes more frequent (19.9 vs 7.2%) a non-contact fall in injured carving skiers compared with injured traditional skiers. However, with the introduction of carving skis, Burtscher et al. [10] showed that the sex-specific knee injury rate remained constant, while the overall injury rate has even decreased by 9%.

Previous studies by Wölfel et al. [20], LaPorte et al. [12] and Rust et al. [19] showed that the most common injury in recreational alpine skiers was an ACL tear. A study by Majewski et al. [33] proved that almost 50% of knee-injured skiers ruptured their ACL. Moreover, with regard to skiing related injuries in general, the most common diagnosis is a tear of the ACL with 15–21% [4].

Unfortunately, these studies [4, 19, 33] do not differentiate between isolated ACL tears and ACL ruptures with concomitant injuries. Therefore, it is difficult to compare these results with the finding of the present study. Since the introduction of the carving skis in the 90s, hardly any study investigated knee injury patterns and provided detailed information about epidemiological aspects.

In their study, Granan et al. [22] investigated knee injury patterns at the time of ACL reconstruction and evaluated activities that led to an ACL injury. About 41% of knee-injured skiers suffered from an isolated ACL injury [22], which seems to be a bit higher compared with findings of the present study, where 36% of skiers reported of isolated ACL injuries.

In the present study, the forward twisting fall (valgus external rotation) was the most frequent reported type of fall among recreational skiers that suffered from an ACL rupture and concomitant injuries. This seems to be in line with the results of other studies by Shea et al. [34], Ruedl et al. [26] and Posch et al. [25] that reported of the valgus external rotation being the most common injury mechanism for recreational skiers in general. In comparison, a study by Järvinen et al. [35] showed an equal distribution between forward twisting fall and backward twisting fall in 32 traditional skiers.

As it was stated in the study by Natri et al. [36], the primary ligament injured in the classic forward twisting fall is usually the medial collateral and the ACL. According to Natri et al. [36], it begins with the skier losing balance with the centre of gravity shifting forward. The inside edge of the ski tip is in contact with the ground and causes an immediate abduction and external rotation of the tibia [36].

In this study, an ACL injury was most often accompanied by an injury to the MCL (50%). Previous studies by Warme et al. [7], Cimino [14] and Duncan et al. [18] have already shown that combined injury to the MCL in skiers with an ACL rupture occurred in 20–57% of cases, well reflecting the underlying results. Interestingly, there seems to be no difference between carving skiers and traditional skiers with regard to the most common concomitant injury patterns. On the other hand, Granan et al. [22] reported that meniscus injuries (43%) were the most common concomitant injuries among knee-injured skiers. The proportion of associated injuries to the MM (40%) seems to be in line with results of studies by Cimino [14], Duncan et al. [18] and Granan et al. [22] that reported the meniscal pathology being associated with an ACL rupture ranged from 23 to 55%. However, in the present study, an injury to the MM (40.1%) was more commonly reported than to LM (20.9%) in recreational skiers with an ACL injury. Prior to the introduction of the carving skis, studies reported of higher proportions of lateral in relation to medial meniscal tears [14, 18]. Potential reason for these differences could be the shift within the type of falls among knee-injured recreational skiers from the phantom foot mechanism (flexion internal rotation) [36] to forward twisting falls [25, 26, 34].

Compared to the study by Cimino [14], who stated injuries to the LCL to be infrequent (3%), in the present study, about 23% of patients suffering from an ACL injury and concomitant injuries injured their LCL. Again, this difference could be potentially explained by the development of the carving skis leading to different, complex injury situations compared to traditional skis. As no PCL injuries were observed and only four patients suffered from cartilage damage, these injury events can be interpreted as rare events. In general, the classic unhappy triad was observed in seven cases. Although no triad of ACL, MCL and LM was seen, Duncan et al. [18] reported this triad occurring 9 times higher than O`Donoghues`s [27] classic unhappy triad.

The majority of ACL injuries were grade III, whereas concomitant injuries (MCL, MM, LCL) were mainly grade II. Group comparisons did not reveal any significant differences in sex, age, BMI, physical fitness, skill level, risk taking behaviour, perceived speed and type of fall between patients with an isolated ACL injury and patients suffering from an ACL injury and concomitant injuries.

Although not reaching statistical significance, more females than males suffered from ACL injuries with concomitant injuries while a contrary sex ratio was found among patients with isolated ACL injuries. Interestingly, patients with ACL injuries and concomitant injuries reported themselves as being predominately more cautious than risky. Again, a contrary situation was found among patients with isolated ACL injuries that reported majoritarian being risky.

Despite the fact that improvements have been taking place in terms of slope preparation, safety standards on ski slopes and creating awareness of potential risk factors, the ACL is predominantly affected, frequently accompanied by injuries of MCL. Several equipment-related factors regarding carving skis such as shorter length, changed flexural and torsional stiffness, enhanced side cut and the increasing distance between the ski boot and snow due to lifters [10] might all contribute to changed knee injury patterns within recreational alpine skiers during the last decades. As the introduction of the carving skis led to different complex injury situations and the forward twisting fall still represents the most frequent type of fall among ACL-injured skiers [25, 34], the ski binding release at the moment of accident potentially plays an important role. A failure of ski binding release means the ski acts as a lever and creates substantial magnitudes of torque about the knee joint for a longer period of time than it would if the binding released [36]. Regular checking the ski bindings by professionals could, therefore, present a potential preventive measure as it was demonstrated that newly adjusted bindings clearly reduced knee injury risk among female skiers [10].

An improved understanding of current knee injury patterns among knee-injured recreational skiers could provide a valuable basis for future research. Potential risk factors must be established before preventive recommendations can be derived. Furthermore, these findings provide useful information to sport physicians and surgeons involved in the diagnosis and treatment of knee injuries in recreational skiers. Due to the fact that almost two-third of the total sample were injuries to the ACL with concomitant injuries, special attention on these injury patterns may enable performing appropriate treatments in a timely manner.

A selection bias of knee-injured recreational skiers due to restriction of our patients to the sportclinic cannot be entirely excluded. However, a major part of skiing related injuries occurring in the study area were treated in the sportclinic and there are no indications of any source of selection. To the best of our knowledge, this is the first study precisely reporting of current knee injury patterns and characteristics of concomitant injuries among recreational alpine skiers (all using carving skis in contrast to earlier studies with traditional skis).

## Conclusion

In conclusion, knee injury patterns in recreational alpine (downhill) skiers have considerably changed since the introduction of carving skis. Whereas, before the introduction of the carving skis, the MCL was reported being the most common injured part of the knee, currently, the majority of knee injuries are ACL injuries accompanied by injury of other knee joint structures, i.e. the MCL, MM and LCL. Most ACL injuries, including isolated and combined ACL injuries, were grade III, indicating a complete rupture whereas concomitant injuries were mainly grade II. Knee injury patterns may reflect the forces associated with complex injury mechanisms. Presented findings may help to better understand injury mechanisms and to improve preventive measures in recreational alpine skiing.
